# The compact mitochondrial genome of *Zorotypus medoensis* provides insights into phylogenetic position of Zoraptera

**DOI:** 10.1186/1471-2164-15-1156

**Published:** 2014-12-21

**Authors:** Chuan Ma, Yeying Wang, Chao Wu, Le Kang, Chunxiang Liu

**Affiliations:** State Key Laboratory of Integrated Management of Pest Insects and Rodents, Institute of Zoology, Chinese Academy of Sciences, Beijing, 100101 China; Laboratory of Systematics and Evolution, Institute of Zoology, Chinese Academy of Sciences, Beijing, 100101 China; Beijing Institutes of Life Science, Chinese Academy of Sciences, Beijing, 100101 China

**Keywords:** Polyneoptera, Embioptera, Long-branch attraction, Gene length reduction, tRNA truncation, Substitution rate

## Abstract

**Background:**

Zoraptera, generally regarded as a member of Polyneoptera, represents one of the most enigmatic insect orders. Although phylogenetic analyses based on a wide array of morphological and/or nuclear data have been performed, the position of Zoraptera is still under debate. Mitochondrial genome (mitogenome) information is commonly considered to be preferable to reconstruct phylogenetic relationships, but no efforts have been made to incorporate it in Zorapteran phylogeny. To characterize Zoraptera mitogenome features and provide insights into its phylogenetic placement, here we sequenced, for the first time, one complete mitogenome of Zoraptera and reconstructed the phylogeny of Polyneoptera.

**Results:**

The mitogenome of *Zorotypus medoensis* with an A + T content of 72.50% is composed of 13 protein-coding genes, 22 transfer RNA genes, 2 ribosomal RNA genes, and a noncoding A + T-rich region. The gene content and arrangement are identical to those considered ancestral for insects. This mitogenome shows a number of very unusual features. First, it is very compact, comprising 14,572 bp, and is the smallest among all known polyneopteran mitogenomes. Second, both noncoding sequences and coding genes exhibit a significant decrease in size compared with those of other polyneopterans. Third, *Z. medoensis* mitogenome has experienced an accelerated substitution rate. Fourth, truncated secondary structures of tRNA genes occur with loss of dihydrouridine (DHU) arm in *trnC*, *trnR*, and *trnS(AGN)* and loss of TΨC arm in *trnH* and *trnT*. The phylogenetic analyses based on the mitogenome sequence information indicate that Zoraptera, represented by *Z. medoensis*, is recovered as sister to Embioptera. However, both Zoraptera and Embioptera exhibit very long branches in phylogenetic trees.

**Conclusions:**

Characterization of *Z. medoensis* mitogenome contributes to our understanding of the enigmatic Zoraptera. Mitogenome data demonstrate an overall strong resolution of deep-level phylogenies of Polyneoptera but not Insecta. It is preferable to expand taxon sampling of Zoraptera and other poorly represented orders in future to break up long branches.

**Electronic supplementary material:**

The online version of this article (doi:10.1186/1471-2164-15-1156) contains supplementary material, which is available to authorized users.

## Background

Zoraptera (angel insects) is one of the most enigmatic insect groups. They are wingless or alate small insects, approximately 3 mm in length [1]. As a hemimetabolous group, these insects resemble termites in morphological appearance and gregarious behavior. They are found worldwide, but mainly have a limited distribution in tropical and subtropical areas. A total of 39 extant species, all of which belong to a single genus *Zorotypus* Silvestri, and 9 extinct species have been described [2] since the order was first described by Silvestri [3]. In China, two species have been recorded in Tibet and one in Taiwan [4–6]. Zoraptera has long been neglected, and only in recent years, morphological, developmental, behavioral, and paleontological knowledge on this species-poor order has been gradually accumulated [2, 6–10].

Zoraptera is considered to be a member of Polyneoptera, a large assemblage of highly diverse insects, including also Blattodea, Dermaptera, Embioptera, Grylloblattodea, Isoptera, Mantodea, Mantophasmatodea, Orthoptera, Phasmatodea, and Plecoptera [1]. The polyneopteran orders represent one of the earliest-branching lineages of insects, so that an insight into their evolutionary relationships can shed light on the early radiation and diversification of insects. Therefore, considerable attention has been attracted to phylogenetic relationships regarding the polyneopteran orders, in particular Zoraptera [11, 12].

Based on morphological characters and/or nuclear gene sequences (e.g. 18S and 28S rDNA), Zoraptera have been proposed to be sister to a wide variety of polyneopteran taxa, including Dermaptera [13–16], Embioptera [1, 17–21], Isoptera [22], Dictyoptera [23–26], Dermaptera + Dictyoptera (Blattodea, Isoptera, and Mantodea) [27], Embioptera + Phasmatodea [7], and Plecoptera + Dermaptera [28]. In addition, transcriptomic data analysis revealed that Zoraptera was sister to the remaining five polyneopteran orders including Blattodea, Dermaptera, Isoptera, Orthoptera, and Plecoptera [29]. A more recent study based on transcriptomic data of eight polyneopteran orders [30] supported that Zoraptera either was sister to Plecoptera or formed the most deeply diverging lineage in Polyneoptera. Meanwhile, non-polyneopteran lineages, such as Acercaria [31, 32], Holometabola [33], and Acercaria + Holometabola [34], were once pointed out to be sister to Zoraptera. However, the hypothesis of its sister-group relationship with the non-polyneopteran lineages was either based on an insufficient evaluation of very incomplete morphological data or an artefact mainly caused by parallel reductions of some of them (e.g. number of Malpighian tubules or tarsomeres) [35]. Therefore, the polyneopteran affinities of Zoraptera are increasingly being accepted. In spite of these efforts, the exact position of Zoraptera within Polyneoptera is far from being resolved and its controversial status has been expressed in terms of “Zoraptera problem” by Beutel and Weide [31].

One major reason for the difficulty in resolving the phylogenetic position of Zoraptera is ancient rapid radiation of Polyneoptera, which diversified almost simultaneously, around 300 million year ago [36]. This results in limited phylogenetic signal left in extant taxa to trace their relationships. In this case, utilization of multiple independent molecular data could offer an important step towards a fully resolved phylogenetic placement of Zoraptera. Mitochondrial DNA (mtDNA) sequences, in particular the whole mitogenome, are increasingly becoming one of the most commonly used markers in resolving insect relationships from intra-specific to inter-ordinal or even higher levels [37–39]. Insect mitogenome sequences deposited in the GenBank database have consequently been increasing rapidly [39], providing an important data source for insect phylogenies. Therefore, mitogenomes have the potential to offer new insights into the placement of Zoraptera. Nevertheless, within Polyneoptera and even Insecta, Zoraptera is the sole order without sequenced complete or nearly complete mitogenomes available in the GenBank database.

In the present study, we sequenced and characterized the complete mitogenome of *Z. medoensis* Hwang, which was described from Motuo County in southeastern Tibet of China [5]. To determine the position of Zoraptera in Polyneoptera, mitogenome sequences of *Z. medoensis* and all other polyneopteran orders as well as six outgroup taxa were used for phylogenetic inferences. This represents the first mitochondrial phylogenomic study encompassing all the eleven orders of Polyneoptera to date. The present study demonstrates an overall strong resolution of deep-level phylogenies of Polyneoptera. Furthermore, to analyze the phylogenetic position of Zoraptera in a broader context, the taxon sampling was enlarged to the class Insecta. However, it is difficult to resolve deep phylogenies of major insect groups with mitogenome datasets.

## Methods

### Sample collection and identification

Samples of *Z. medoensis* were collected from the type locality of the species in Motuo County (29.37°N, 95.13°E) in southeastern Tibet of China. They were preserved in 100% ethanol and maintained at 4°C until used. Besides examination and comparison of their external morphology to that of the types of *Z. medoensis* [5], molecular evidence was also provided to confirm the identity of the species used in our study. A 1.5-kb fragment of 18S rDNA and a 2.4-kb fragment of 28S rDNA were amplified (primers were provided in Additional file 1) and sequenced as below. Both sequences were deposited in GenBank under accession numbers KM246626 and KM246627. A BLASTN search against the nucleotide collection in GenBank indicated that the two rDNA sequences had the highest identity to those of other *Zorotypus* species, corroborating the identity of *Z. medoensis* we used. These two rDNA sequences were not included in our phylogenetic analyses because such sequences of other Zoraptera species had been utilized in previous studies [14–16, 23, 25, 26, 28].

### Mitogenome amplification and sequencing

Total DNA extracted from a single *Z. medoensis* specimen with the DNeasy Blood & Tissue kit (Qiagen) was used as templates for subsequent PCR amplification. Five pairs of primers (Additional file 1) were designed to amplify overlapping fragments ranging from 1.8 kb to 5.1 kb that covered the whole mitogenome. The PCR reactions were performed using LA Taq™ (TaKaRa Co., Dalian, China) with the following cycling conditions: 95°C for 1 min; 30 cycles of 98°C for 10 s, 55°C for 10 s, and 68°C for 2–5 min (1 min for 1-kb products); 68°C for 10 min. The purified PCR products were sequenced via 3730×L DNA Analyser (Applied Biosystems). The resulting mtDNA sequences were assembled using SeqMan implemented in the Lasergene software (DNAStar, Inc.).

### Mitogenome annotation

Mitogenome annotation was performed using the MITOS web server [40]. All tRNA genes were identified and folded into secondary structures by the MITOS, with the exception of *trnF*, which was identified by the online tRNAscan-SE search server using invertebrate mitochondrial codon predictors [41]. For *trnI*, the predicted secondary structures from the two programs were different, i.e. a G-C pair as the TΨC arm in the tRNAscan-SE whereas an unpaired stretch in the MITOS. Here, we adopted the structure from the tRNAscan-SE, as a single G-C pair was designated as the TΨC arm in *trnC* and *trnL(CUN)* by the MITOS. Although all the protein-coding genes (PCGs) and rRNA genes were identified by the MITOS, most gene boundaries remained unresolved, as was evidenced by either lack of typical start or stop codons or presence of long intergenic spacers. Such gene boundaries were determined by alignment of homologous genes in Polyneoptera. In cases when a stop codon of PCGs, with the exception of *atp8* and *nad4L*, was located in downstream genes encoded by the same strand, an incomplete stop codon (T or TA) was designated to avoid gene overlaps [42].

### Bioinformatic analyses

Nucleotide composition of *Z. medoensis* mitogenome was calculated using DAMBE [43]. AT-skew [(A − T)/(A + T)] and GC-skew [(G − C)/(G + C)] were used to measure nucleotide compositional differences. Nucleotide substitution saturation for each of the three codon positions of PCGs was assessed by DAMBE [43, 44]. Codon usage and the relative synonymous codon usage (RSCU) values were calculated using MEGA5 [45]. PHYLTEST [46], which allowed the two groups for comparison to contain multiple taxa, was used to conduct relative-rates test between Zoraptera and each of all other polyneopteran orders. This test was based on the DNA datasets (PCG123RNA and PCG12RNA; see below) with Kimura 2-parameter and the protein dataset (PCG-AA) with Poisson correction method.

Student’s one-sample *t*-test was conducted using the SPSS 20.0 software (IBM, Chicago, IL, USA) to compare gene/region lengths between Zoraptera and all other available polyneopterans with complete mitogenomes. The mitogenome information for this analysis was listed in Additional file 2. Note that all 22 tRNA genes, due to their short lengths, were concatenated as a single alignment. The A + T-rich region referred to the non-coding sequence between *rrnS* and *trnI* in all polyneopterans, except *Sinochlora longifissa* where this region was translocated downstream of *nad2* [47]. Although two A + T-rich regions were present in *Ramulus hainanense*, only the one adjacent to *rrnS* was used.

### Phylogenetic analyses

The taxa for phylogenetic analyses of Polyneoptera were listed in the Additional file 2. Only one complete mitogenome had been sequenced for each of four polyneopteran orders including Dermaptera, Mantodea, Mantophasmatodea, and the currently sequenced Zoraptera. Embioptera and Grylloblattodea each had one nearly complete mitogenome available. For the five other orders that had multiple complete or nearly complete mitogenomes sequenced, one representative from each family was selected in the present study to save computational time and to better represent taxonomic diversities. This led to an inclusion of 2, 4, 5, 6, and 18 mitogenomes from Plecoptera, Blattodea, Phasmatodea, Isoptera, and Orthoptera, respectively. Together, the resulting sampling included a total of 41 polyneopteran mitogenomes, representing all orders in Polyneoptera. To root the phylogenetic tree of Polyneoptera, 6 outgroup taxa from Zygentoma (1 species), Archaeognatha (1 species), Ephemeroptera (2 species), and Odonata (2 species) were added in the analysis (Additional file 2). This outgroup selection was based on previous phylogenetic relationships [11, 48].

GenBank files of all the 47 mitogenomes were downloaded from the GenBank database and used to extract each of the 37 gene and 13 protein sequences using the Perl scripts [49]. The 22 tRNA genes, 2 rRNA genes, and 13 protein sequences were each aligned using the web server of MUSCLE [50]. The 13 PCG nucleotide sequences were retro-aligned using the corresponding amino acid alignment as implemented in PAL2NAL [51] with the invertebrate mitochondrial code table. A few tRNA genes that were not sequenced in incomplete mitogenomes were coded as missing data. Poorly aligned positions and divergent regions were deleted by the Gblocks server [52] with a more stringent selection, i.e. do not allow many contiguous non-conserved positions. The DNA and protein sequences were separately concatenated to obtain final datasets for phylogenetic analyses.

Two DNA datasets i.e. PCG123RNA (all codon positions of 13 PCGs plus all tRNA and rRNA genes) and PCG12RNA (1st + 2nd codon positions plus all tRNA and rRNA genes) were built. Substitution models and optimal partitioning schemes were selected under the Bayesian Information Criterion (BIC) using greedy search algorithms and unlinked branch lengths in PartitionFinder [53]. The partitions (42 for PCG123RNA and 29 for PCG12RNA) we pre-defined before running the PartitionFinder included individual codon position of each PCG, concatenated tRNA genes, the *rrnL* gene, and the *rrnS* gene. Among these partitions, the best partitioning strategies and their respective substitution models simultaneously identified by PartitionFinder were used in subsequent phylogenetic reconstructions.

Bayesian Inference (BI) and Maximum Likelihood (ML) analyses were conducted based on each of the two partitioned DNA datasets using MrBayes v3.2 [54] and RAxML v7.2.6 [55], respectively. For the BI analysis, two runs of one million generations using 24 Markov chains were carried out, sampling every 100 generations. All model parameters (statefreq, revmat, shape, and pinvar) were unlinked to make sure that each partition had its own set of parameters. “Ratepr = variable” was set to allow all partitions to evolve under different rates. The first 25% of the trees were discarded as burn-in, and the remaining were used to generate a 50% majority rule consensus tree with nodal confidence assessed with posterior probabilities (BPP). Convergence of the chains was assessed by the standard deviation of the split frequencies (lower than 0.01). For the ML analysis, the GTRGAMMA model was used and 200 ML searches were executed with a random starting tree and a rapid hill-climbing algorithm (−f d). The tree topology with the best likelihood score was selected. Bootstrap values (BS) were calculated via 1000 replicates estimated in RAxML.

In addition, one protein dataset PCG-AA, i.e. concatenation of 13 protein sequences, was also used for phylogenetic analysis as implemented in PhyloBayes 3.3 [56]. The site-heterogeneous mixture model of CAT [57], which could account for among-site heterogeneity and lessen the influences of long-branch attraction (LBA), was used. Two replicates were run and at least 10,000 samples were drawn from the posterior. The analysis was stopped until the maximum discrepancy in frequencies of bipartitions was below 0.3, which indicated that the samples had given a good qualitative picture of the posterior consensus [56].

Finally, despite the growing body of evidence for the polyneopteran affinities of Zoraptera, opposing views have also been proposed (see the Background). To examine these possibilities, we enlarged the taxon sampling to the class Insecta by including protein sequences of Holometabola and Acercaria (Additional file 2). A total of 9 and 2 species representing Holometabola and Acercaria, respectively, were added. Some orders (e.g. Hymenoptera, Strepsiptera, Psocoptera, Phthiraptera, and Thysanoptera) exhibiting long branches in previous studies [58] and in our initial analyses (results not shown) were excluded. The protein dataset (Insecta-AA) was prepared and used as above mentioned for phylogenetic reconstruction with PhyloBayes.

### Likelihood-mapping analysis

To investigate phylogenetic signals contained in each of the datasets (PCG12RNA, PCG123RNA, and PCG-AA), we conducted a likelihood-mapping analysis of 10,000 random quartets using TREE-PUZZLE [59, 60]. A likelihood map consists of an equilateral triangle containing dots representing the likelihoods of the three possible unrooted trees for a set of four sequences (quartets) randomly selected from the dataset. The dots near the corners or at the sides respectively represent tree-like (fully resolved phylogenies in which one tree is better than the others) or network-like phylogenetic signals (three regions in which it is not possible to decide between two topologies); the dots at the central area of the map represents star-like signals (the region where the star tree is optimal).

### Tree topology test

The topology test based on PCG12RNA was performed to compare Zoraptera + Embioptera and other major alternative hypotheses [30], i.e. Zoraptera + Dictyoptera, Zoraptera + Dermaptera, Zoraptera + Plecoptera, and Zoraptera + all other polyneopteran orders. The per-site log likelihood scores were calculated for the best-scoring ML trees under each constrained topology using the -f g option in RAxML. The outputs were submitted to CONSEL ver. 0.1i [61] to make a statistical comparison.

## Results

### Characterization of *Z. medoensis*mitogenome

The complete mitogenome of *Z. medoensis* (GenBank accession number KJ467512) comprised 13 PCGs, 2 rRNA and 22 tRNA genes, and a non-coding A + T-rich region (Table 1), all of which were arranged in the same order considered ancestral for insects [39]. The mitogenome was 14,572 bp in length, representing the shortest of polyneopteran mitogenomes sequenced so far. Nucleotide composition calculation (Additional file 3) showed a strong preference for A and T, which accounted for 72.50% in *Z. medoensis* mitogenome.

**Table 1:** **Annotation of** ***Zorotypus medoensis*** **mitogenome**

Gene/region	Strand*	Start	Stop	Start codon	Stop codon	Anticodon	Intergenic spacer
*trnI*	J	1	60			GAT	-
*trnQ*	N	58	126			TTG	−3
*trnM*	J	126	187			CAT	−1
*nad2*	J	188	1163	ATT	T––		0
*trnW*	J	1164	1221			TCA	0
*trnC*	N	1214	1260			GCA	−8
*trnY*	N	1260	1322			GTA	−1
*cox1*	J	1327	2854	CGA	T––		4
*trnL(UUR)*	J	2855	2915			TAA	0
*cox2*	J	2916	3576	ACG	T––		0
*trnK*	J	3577	3636			CTT	0
*trnD*	J	3636	3695			GTC	−1
*atp8*	J	3696	3848	ATC	TAA		0
*atp6*	J	3842	4500	ATG	TA-		−7
*cox3*	J	4501	5281	ATG	T––		0
*trnG*	J	5282	5339			TCC	0
*nad3*	J	5340	5688	ATA	T––		0
*trnA*	J	5689	5747			TGC	0
*trnR*	J	5747	5797			TCG	−1
*trnN*	J	5795	5856			GTT	−3
*trnS(AGN)*	J	5854	5909			GCT	−3
*trnE*	J	5909	5966			TTC	−1
*trnF*	N	5955	6013			GAA	−12
*nad5*	N	6014	7697	ATT	T––		0
*trnH*	N	7698	7755			GTG	0
*nad4*	N	7756	9085	ATG	T––		0
*nad4L*	N	9079	9360	ATG	TAA		−7
*trnT*	J	9363	9417			TGT	2
*trnP*	N	9418	9476			TGG	0
*nad6*	J	9479	9928	ATA	TAA		2
*cob*	J	9932	11039	ATG	T––		3
*trnS(UCN)*	J	11040	11102			TGA	0
*nad1*	N	11120	12049	ATT	TAG		17
*trnL(CUN)*	N	12050	12107			TAG	0
*rrnL*	N	12108	13301				0
*trnV*	N	13302	13361			TAC	0
*rrnS*	N	13362	14085				0
A + T-rich region	J	14086	14572				0

*J, majority strand; N, minority strand.

The *Z. medoensis* mitogenome possessed gene overlaps and intergenic spacers, with the former more common than the latter (Table 1). A total of 12 overlaps (48 bp in total length) between genes were present in *Z. medoensis*. A 7-bp overlap of *atp8*-*atp6* and a 7-bp overlap of *nad4L*-*nad4* existed, which were usually found in other insect mitogenomes [42, 62]. Except *trnT*-*trnP*, all adjoining tRNA genes, no matter whether they were encoded by the same strand or not, had overlaps. The largest overlap was 12 bp located between *trnE* and *trnF*. Five intergenic spacers, totaling 28 bp, were detected, with the largest one (17 bp) located between *trnS(UCN)* and *nad1*.

Eleven of the 13 PCGs started with the typical codon ATN. However, for *cox1* and *cox2*, the putative initiation codon was CGA and ACG, respectively, both of which were designated in previous studies [63–65]. In addition to canonical stop codons (TAG for *nad1* and TAA for *atp8*, *nad4L*, and *nad6*), incomplete codons (T or TA) were proposed for the other PCGs immediately followed by a tRNA gene on the same strand. The 13 PCGs consisted of 3,623 codons, excluding stop codons. AUU (Ile; 317) and GCG (Ala; 3) were the most and least used codons, respectively. The most abundant codon family was Ile, followed by Leu(UUR) and Met, whereas the least abundant codon family was Cys. Compared with the first (68.34%) and second (67.57%) codon positions, the third codon positions showed an even stronger bias toward A + T (77.73%) (Additional file 3). RSCU analysis indicated that all two-fold degenerate codons were A + T biased in the third codon positions. It was also the same case for four-fold degenerate codons except Gly and Arg, both of which had a preference of A + G to C + T.

*Z. medoensis* mitogenome contained a total of 22 tRNA genes, which ranged from 47 bp (*trnC*) to 69 bp (*trnQ*) in size. Of them, 17 could be folded into typical cloverleaf secondary structures (Additional file 4). The lack of dihydrouridine (DHU) arm was found in *trnC*, *trnR*, and *trnS(AGN)*, while the absence of TΨC arm occurred in *trnH* and *trnT*. The lack of DHU arm in *trnS(AGN)* is very common in insect mitogenomes [42, 48, 62], whereas the occurrence of truncated secondary structures of other tRNA genes was limited and was reported e.g. in Protura [66].

The *rrnL* and *rrnS* genes were 1,194 bp and 724 bp, respectively. The A + T content was 75.38% for *rrnL* and 75.14% for *rrnS* (Additional file 3). The 487-bp A + T-rich region, located between *rrnS* and *trnI*, had an A + T content of 76.18%, lower than the average value of tRNA genes (77.70%). Tandem repeat units larger than 50 bp, often observed in insect mitogenomes, were not detected within the A + T-rich region of *Z. medoensis*.

### Comparison of gene/region lengths

**Figure 1:**
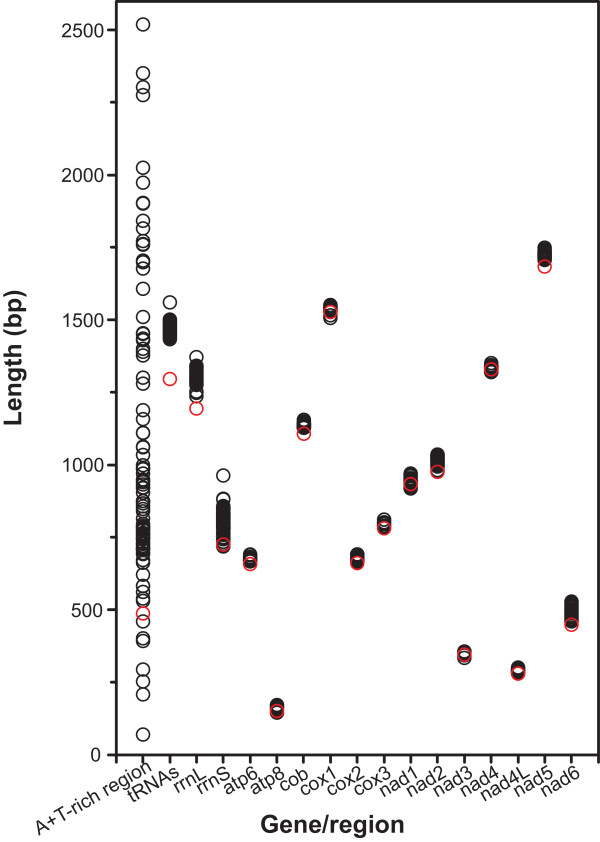
**Sequence lengths of mitochondrial genes and the A + T-rich region.** Polyneopteran mitogenomes included are listed in Additional file 2. Red circles refer to values of *Zorotypus medoensis*. The values for tRNAs are those of all concatenated tRNA genes from each mitogenome. For PCGs, stop codons are excluded.

To identify sources contributing to the reduced mitogenome size, sequence lengths of major genes/regions between *Z. medoensis* and all other complete polyneopteran mitogenomes were compared and illustrated in Figure 1. Sizes of the A + T-rich region were most variable, ranging from 69 bp in orthopteran *Ruspolia dubia* to 2,519 bp in phasmatodean *Megacrania alpheus adan*. The A + T-rich region of *Z. medoensis* was 487 bp, statistically shorter than those of all other polyneopteran mitogenomes sequenced to date (mean value = 1,024 bp; Student’s one-sample *t*-test, *P* < 0.001). In contrast, all coding genes were overall conserved due to functional constraints; however, all PCGs, *rrnS*, *rrnL*, and concatenated tRNA genes were significantly shorter in *Z. medoensis* than other polyneopterans (*P* < 0.001 for all). Notably, concatenated tRNA genes in *Z. medoensis* were 1,296 bp in size, which were 11.96% shorter than the average size (1,472 bp) of all other polyneopteran counterparts. For *rrnS* and *rrnL* in *Z. medoensis*, the size reduction was 10.06% and 8.86%, respectively. Conversely, PCG size variations were overall minimal.

### Saturation test and nucleotide bias calculation

**Figure 2:**
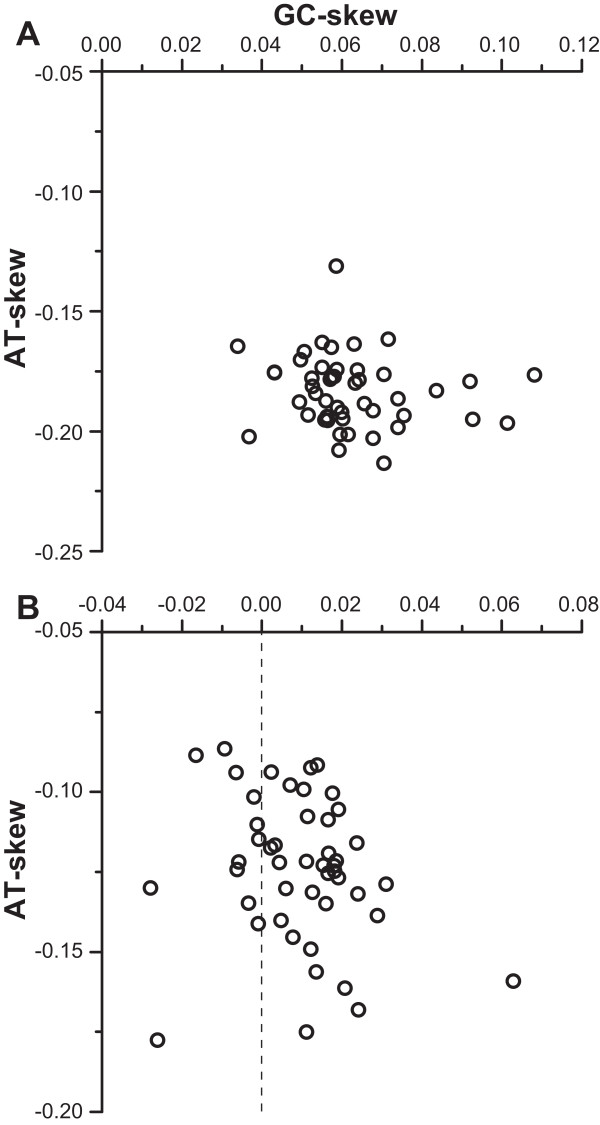
**Scatter plot of AT- and GC-skews for the datasets of PCG12RNA (A) and PCG123RNA (B).**

Saturation test of the third codon positions of concatenated PCGs indicated that the index of substitution saturation, Iss, was significantly higher than the critical value of the index of saturation, Iss.cAsym (*P* < 0.001; NumOUT = 16 or 32). This result suggested that these positions experienced so much saturation that they were useless for phylogenetic reconstruction [44]. Furthermore, the AT- and GC-skew calculation (Figure 2) indicated that inclusion of the third codon positions induced nucleotide compositional bias. Specifically, all taxa exhibited negative AT- and positive GC-skews for the dataset of PCG12RNA; in contrast, for PCG123RNA, GC-skew values turned negative for 12 species.

### Mitogenome-based phylogeny

To unravel the phylogenetic status of Zoraptera in Polyneoptera, phylogenetic analyses were conducted based on mitogenome datasets (PCG12RNA, PCG123RNA, and PCG-AA) from 41 polyneopteran species, representing all the 11 orders of Polyneoptera, and 6 outgroup species. The likelihood-mapping analysis showed that more than 99.4% of the randomly chosen quartets fell in the corners (Additional file 5), thus indicating that each of the three datasets contained sufficient phylogenetic information.

**Figure 3:**
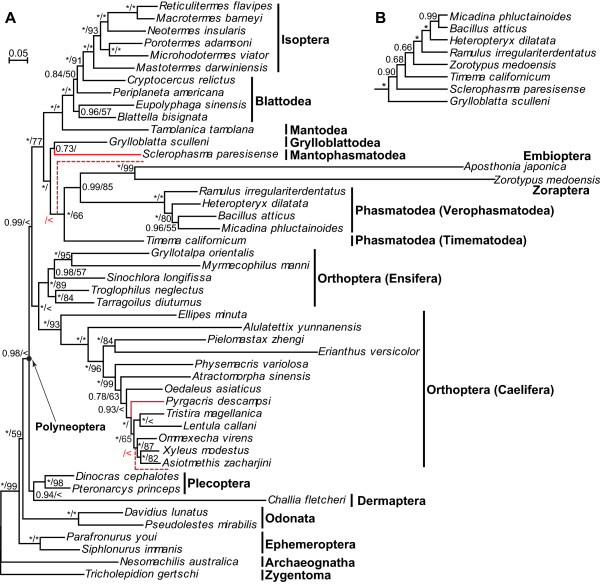
**Phylogenetic trees of Polyneoptera based on the dataset of PCG12RNA. (A)** Bayesian phylogram of Polyneoptera. The numbers at the nodes refer to Bayesian posterior probabilities (BPP) and ML bootstrap support (BS) values. Nodal support values of 1.00 for BPP or 100% for BS are represented by “*”; BS values below 50% are represented by “ < ”. The solid red branches are not supported by the ML method; their positions under the ML method are indicated by dashed red branches with BS shown around. The dashed red branches are not scaled to their lengths. **(B)** Bayesian cladogram after removing *Aposthonia japonica* (Embioptera). Only a partial tree is displayed because the other branching patterns remain invariable.

The BI and ML tree topologies inferred from PCG12RNA were largely concordant (Figure 3A). The monophyletic Polyneoptera was recovered (BPP = 0.98, BS < 50%). Zoraptera, represented by *Z. medoensis*, was sister (BPP = 1.00, BS = 99%) to Embioptera, represented by *Aposthonia japonica*. Zoraptera + Embioptera had a close relationship (BPP = 0.99, BS = 85%) to Verophasmatodea of Phasmatodea, and these taxa formed a sister group (BPP = 1.00, BS = 66%) with Timematodea of Phasmatodea, indicating a paraphyly of Phasmatodea. Plecoptera and Dermaptera clustered as a sister group (BPP = 0.94, BS < 50%), which split first within Polyneoptera. The two suborders (Caelifera and Ensifera) of Orthoptera were each strongly recovered as monophyletic, and further constituted a monophyletic Orthoptera (BPP = 1.00, BS < 50%). Blattodea was paraphyletic with two species more close to the monophyletic Isoptera (BPP = 1.00, BS = 100%). The assemblage of Blattodea and Isoptera was sister (BPP = 1.00, BS = 100%) to Mantodea, strongly supporting the monophyly of Dictyoptera. The position of Mantophasmatodea was not stable, either sister to Grylloblattodea with a BPP value of 0.73 or to Zoraptera + Embioptera + Phasmatodea with a BS value of less than 50%.

**Figure 4:**
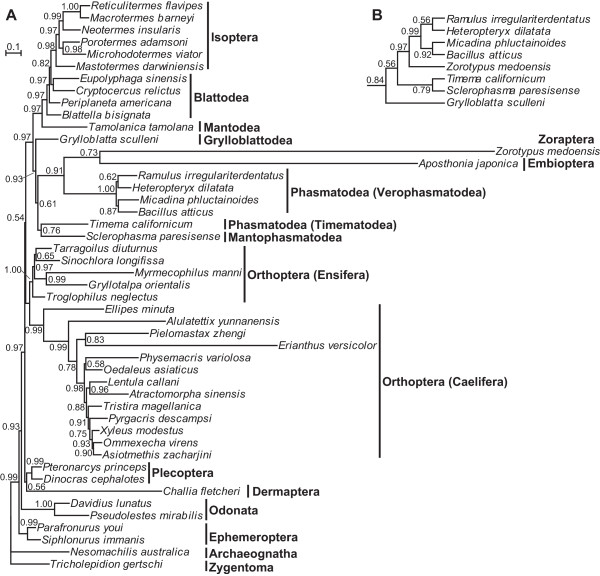
**Bayesian trees inferred from concatenated protein sequences (PCG-AA) of Polyneoptera. (A)** Bayesian phylogram of Polyneoptera with BPP values indicated at nodes. **(B)** Bayesian cladogram after removing *Aposthonia japonica* (Embioptera). As the resulting tree topology remains unchanged, only Zoraptera and closely related taxa are shown.

The tree topology inferred from the protein dataset (PCG-AA) further supported the sister-group relationship (BPP = 0.73) of Zoraptera and Embioptera (Figure 4A). However, both taxa still had long branches. The tree topology was, at the inter-ordinal level, congruent with that based on PCG12RNA with the exception that Mantophasmatodea was sister (BPP = 0.76) to Timematodea of Phasmatodea.

To evaluate phylogenetic performance of the third codon positions, we included them (i.e. using the dataset of PCG123RNA) and reconstructed the phylogeny (Additional file 6). The resulting tree strongly supported the sister-group relationship (BPP = 1.00, BS = 100%) between Zoraptera and Embioptera with long branches for both taxa. Compared with the results based on PCG12RNA, the tree topology inferred from PCG123RNA varied mainly for the following orders (Additional file 6). Polyneoptera was not recovered as monophyletic due to the insertion of Ephemeroptera, which was sister (BPP = 0.96, BS < 50%) to Dermaptera. For Orthoptera, the two monophyletic suborders (Caelifera and Ensifera) did not group together, thus rejecting the monophyly of Orthoptera. Mantophasmatodea was supported as the sister (BPP = 0.99, BS < 50%) of Grylloblattodea.

To test whether LBA affected our phylogenetic inferences, BI analyses using PCG12RNA and PCG-AA were each re-conducted after exclusion of Embioptera. The resulting trees supported the sister clade of Zoraptera and Verophasmatodea of Phasmatodea (Figures 3B and 4B). The BPP value was 0.66 for the dataset PCG12RNA and 0.97 for PCG-AA. In addition, the topology test indicated that Zoraptera + Embioptera represented the most likely phylogeny and other hypotheses were confidently rejected (Table 2).

**Table 2:** **Statistical comparisons among alternative sister-taxa of Zoraptera from major hypotheses**

Sister-taxa of Zoraptera	Log-likelihood	AU	NP	BP	PP	KH	SH	WKH	WSH
Embioptera	−147987.294	0.989	0.984	0.984	1.000	0.998	0.999	0.986	0.999
Dictyoptera	−148041.775	0.003	0.001	0.001	<0.001	0.002	<0.001	0.002	0.003
Dermaptera	−148043.684	0.018	0.015	0.015	<0.001	0.014	<0.001	0.014	0.025
All other Polyneoptera	−148050.515	0.001	<0.001	<0.001	<0.001	<0.001	<0.001	0.001	0.003
Plecoptera	−148052.927	<0.001	<0.001	<0.001	<0.001	<0.001	<0.001	0.001	0.001

Statistical tests include the approximately unbiased test (AU), bootstrap probability (NP and BP), Bayesian posterior probability (PP), Kishino-Hasegawa test (KH), Shimodaira-Hasegawa test (SH), weighted Kishino-Hasegawa test (WKH), and weighted Shimodaira-Hasegawa test (WSH).

The branches of *Z. medoensis* and *A. japonica* in each analysis were extremely long, possibly suggesting considerably accelerated substitution rates of their mitogenomes. The relative-rates test (Additional file 7) confirmed that Zoraptera evolved significantly faster than all other polyneopteran orders except Embioptera, with either Ephemeroptera or Odonata set as outgroup for PCG12RNA and PCG-AA. For PCG123RNA, similar results were obtained with Ephemeroptera as outgroup, whereas Dermaptera also exhibited a faster substitution rate when Odonata was selected as outgroup.

To test the possibilities of sister-group relationships between Zoraptera and non-polyneopterans, we enlarged the taxon sampling to the class Insecta by adding Holometabola and Acercaria. Overall, the BS values for major nodes were relatively low (Additional file 8). The sister-group relationship between long-branched Zoraptera and Embioptera was supported (BPP = 0.92). The two hemipteran species representing Acercaria formed a sister clade. The monophyly of Holometabola was recovered (BPP = 0.75), but Polyneoptera was not supported as monophyletic.

## Discussion

### Mitogenome compactness

*Z. medoensis* mitogenome, although possessing the same gene content as the ancestral insect mitogenome [39], is the shortest polyneopteran mitogenome sequenced to date. On a broad scale, it is only larger than proturan S*inentomon erythranum* (14,491 bp) [66], hemipteran *Neomaskellia andropogonis* (14,496 bp) [67], and dipteran *Rhopalomyia pomum* (14,503 bp) [68] among all currently available complete mitogenomes of hexapods excluding Psocodea (booklice, barklice, and true lice), which generally have multiple minicircular mitogenomes [69].

Our analyses reveal that both noncoding sequences and coding genes contribute to the size reduction of *Z. medoensis* mitogenome (Figure 1). Generally, size variation of insect mitogenomes is attributed to the highly variable-sized noncoding A + T-rich region and/or intergenic spacers [42, 70]. For *Z. medoensis*, the small A + T-rich region (487 bp), which is shorter than half of the average size (1,024 bp) of other polyneopteran mitogenomes, and the short intergenic spacers (28 bp in total), account in part for the compactness. On the other hand, all the coding genes of *Z. medoensis* also have a size reduction. Particularly, concatenated tRNA genes display a size reduction of 11.96%. For some tRNA genes, the size reduction is reflected by loss of TΨC or DHU arms (Additional file 4). These results are consistent with the proposed association between mitogenome size reduction and gene length shortening [71]. From an evolutionary perspective, smaller mitogenome sizes are selectively favored due to a replication advantage and a slower rate of slightly deleterious mutation accumulation [71].

### Accelerated substitution rate

The relative-rates test reveals that *Z. medoensis* mitogenome has a significantly accelerated substitution rate relative to all other polyneopteran orders except Embioptera as well as Dermaptera in one case (Additional file 7). The extremely long branch of *Z. medoensis* in each phylogenetic tree provides additional evidence for its fast substitution rate. Accelerated rates of arthropod mitogenomes have been suggested to be caused by three main factors, i.e. mitogenome rearrangements, parasitic lifestyle, and small body size [72]. In addition, metabolic rate, generation time, and environmental temperature are also correlated with substitution rate variations [73, 74]. These factors are not independent to one another; for example, body size and temperature could affect metabolic rate [75] and generation time [76]. For Embioptera, the fast-evolving mitogenome of *A. japonica* was primarily attributed to frequent gene rearrangement [77]. For *Z. medoensis*, a non-parasitic insect, there is no rearrangement in the mitogenome. Among all the factors listed above, the small body size of *Z. medoensis*, less than 4 mm [5], and/or warm environments they are living in may explain the fast substitution rate. However, Thomas et al. [78] found no evidence of body size influence on invertebrate substitution rates. We should note that their study included, for insects, only two holometabolous orders (Lepidoptera and Hymenoptera) and three mitochondrial genes (*cox1*, *cox2*, and *nad5*), so the body size effect for Polyneoptera cannot be ruled out. It is possible that other as yet undiscovered factors also contribute to the fast substitution rate of *Z. medoensis* mitogenome.

### Phylogeny

In our current taxon sampling, the third codon positions of PCGs are severely saturated and cause biased nucleotide composition (Figure 2). The inclusion of the third codon positions for the phylogenetic reconstruction results in the polyphyly of Orthoptera and the placement of one outgroup order (Ephemeroptera) within Polyneoptera (Additional file 6), contradicting with widely accepted hypotheses [1, 11]. Such saturation and compositional heterogeneity are particularly misleading in phylogenetic inferences [44, 79] and third codon positions have been suggested to be excluded from analyses of deep-level phylogenies [62, 72, 80, 81]. Our results strengthen the idea that the third codon positions with saturation and compositional heterogeneity should be excluded from mtDNA-based phylogenies of Polyneoptera. Therefore, the datasets of PCG12RNA and PCG-AA could better reflect the true evolutionary history.

Zoraptera, whose mitogenome data have never been included before, is consistently recovered as sister to Embioptera in all our phylogenetic analyses, supporting the clade of Mystroptera (= Zoraptera + Embioptera) [21]. Their close affinities were further corroborated by the topology test (Table 2). The clade of Zoraptera + Embioptera was first proposed by Minet and Bourgoin [82] and was further supported by recent morphological studies [17–21]. Our study provides, for the first time, molecular evidence for their sister-group relationship.

In earlier phylogenetic studies involving Zoraptera, very limited molecular markers were frequently used, including only 18S and 28S rDNA, Histone 3 DNA sequence, and three nuclear-encoded protein sequences [14–16, 23, 25, 26, 28]. The resolving power of phylogenetic analyses based on a small number of genes is generally weak due to a restricted amount of phylogenetic information in individual genes. The situation is even worse for Polyneoptera, which has experienced ancient rapid radiation [36]. In addition, some analytical methods in previous studies were pointed out to be problematic [83]. These factors resulted in generally low nodal supports, leaving the position of Zoraptera unstable and controversial. Recent high-throughput sequencing techniques promote accumulation of transcriptomic data and such data have begun to be applied in Polyneoptera [29, 30, 84]. Although the large datasets demonstrated an overall strong resolving power, the position of Zoraptera was inconsistent and generally weakly supported [30]. Therefore, in the context of poor sampling with one representative for most of the eight polyneopteran orders available, current transcriptomic data cannot conclusively address the position of Zoraptera. Here, based on mitogenome datasets, we provide further evidence for the placement of Zoraptera as sister to Embioptera, although a dense sampling has yet to be achieved to solidify the grouping. It should be noted that, when the taxon sampling was enlarged to Insecta, the resulting tree shows an overall poor statistical supports for major deep nodes (Additional file 8), possibly due to the limits of mitogenome datasets in resolving deep phylogeny of Insecta [38, 58]. Taken together, mitogenome data demonstrate an overall strong resolution of deep-level phylogenies of Polyneoptera but not Insecta.

In our study both Zoraptera and Embioptera exhibit rather long branches (Figures 3A and 4A, Additional file 6 and Additional file 8) due to accelerated substitution rates (Additional file 7). Such long branches may be grouped together due to LBA artefacts rather than true relatives. LBA refers to erroneous clustering of long branches as sister groups caused by methodological artefacts [81]. To avoid LBA effects, many strategies have been proposed [57, 81], such as omitting faster evolving characters (e.g. third codons of PCGs), removing long-branch taxa, enlarging taxon sampling to break up long branches, and adopting the CAT model. In our study, either excluding third codon positions or using more conserved protein sequences with the CAT model still generates long branches for both Zoraptera and Embioptera (Figures 3A and 4A). After removing Embioptera, the position of Zoraptera remains unchanged (Figures 3B and 4B). Therefore, the LBA effect on the phylogenetic position of Zoraptera appears not so obvious, but at present it cannot be ruled out. Similarly, a mitogenome-based phylogenetic study of Polyneoptera without Zoraptera and Dermaptera also demonstrated a long-branch Embioptera, but no LBA effect was detected [77]. To break up long branches, it is crucial to expand taxon sampling of both Zoraptera and Embioptera. Furthermore, for Dermaptera, Mantodea, Mantophasmatodea, Grylloblattodea, and Timematodea of Phasmatodea, which are each poorly represented by only a single species with mitogenome information available, intensive taxon sampling is also highly desirable. With increased taxon sampling densities, selection and usage of mitogenomes from those taxa without showing long branches can greatly improve phylogenetic accuracy of Polyneoptera.

## Conclusions

The mitogenome of *Z. medoensis*, the first representative of Zoraptera, shows a number of very unusual features, including compactness, tRNA truncation, and fast substitution rate. Phylogenetic analyses strongly support the sister-group relationship of Zoraptera and Embioptera, offering molecular evidence for the first time to corroborate previous morphological results. Characterization of *Z. medoensis* mitogenome adds on our knowledge not only of the enigmatic Zoraptera but also of early diversification of insects. To improve phylogenetic accuracy, mitogenome sequencing of extensive taxon sampling of Zoraptera and Embioptera as well as other currently sparsely represented orders (e.g. Dermaptera, Mantodea, Mantophasmatodea, Grylloblattodea, and Timematodea of Phasmatodea) is preferable.

### Availability of supporting data

The datasets supporting the phylogenetic results of this article are available in the TreeBASE (http://purl.org/phylo/treebase/phylows/study/TB2:S16788).

## Electronic supplementary material

Additional file 1: **Primer sequences used for PCR amplification.** (XLSX 11 KB)

Additional file 2: **Mitogenome information.** The mitogenomes for gene/region length comparison and phylogenetic analyses are indicated. (XLSX 21 KB)

Additional file 3: **Nucleotide compositions (in percentage).** (XLSX 10 KB)

Additional file 4: **Secondary structures of 22 tRNAs.** (PDF 1 MB)

Additional file 5: **Likelihood map of PCG12RNA (A), PCG123RNA (B), and PCG-AA (C).** The numbers indicate the percentage of dots. (PDF 262 KB)

Additional file 6: **Bayesian phylogram of Polyneoptera based on PCG123RNA.** The numbers at the nodes refer to BPP and BS values. Nodal support values of 1.00 for BPP or 100% for BS are represented by “*”; BS values below 50% are represented by “ < ”. The solid red branches are not supported by the ML method; their positions under the ML method are indicated by dashed red branches with BS values shown around. The dashed red branches are not scaled to their lengths. (PDF 444 KB)

Additional file 7: **Relative-rates test as implemented in PHYLTEST.** (XLS 40 KB)

Additional file 8: **Bayesian phylogram inferred from concatenated protein sequences of Insecta (Insecta-AA).** Branches with BPP values below 50% are collapsed. For visual clarity, Polyneoptera, Acercaria, and Holometabola are marked with red, green, and blue colors, respectively. (PDF 453 KB)

## Abbreviations

*atp6* and *atp8*: ATP synthase subunits 6 and 8
*cob*: Cytochrome b
*cox1*–*3*: Cytochrome c oxidase subunits 1 to 3
*nad1*–*6* and *nad4L*: NADH dehydrogenase subunits 1 to 6 and 4 L
*rrnS* and *rrnL*: Small and large ribosomal RNA (rRNA) subunits
*trnX*: Transfer RNA genes where X is the one-letter abbreviation of the corresponding amino acid.
