# Supplementary figures and images for: The compact mitochondrial genome of Zorotypus medoensis provides insights into phylogenetic position of Zoraptera
Source: BMC Genomics. 2014 Dec 21;15(1):1156. doi: 10.1186/1471-2164-15-1156 (PMC4367826; doi:10.1186/1471-2164-15-1156)

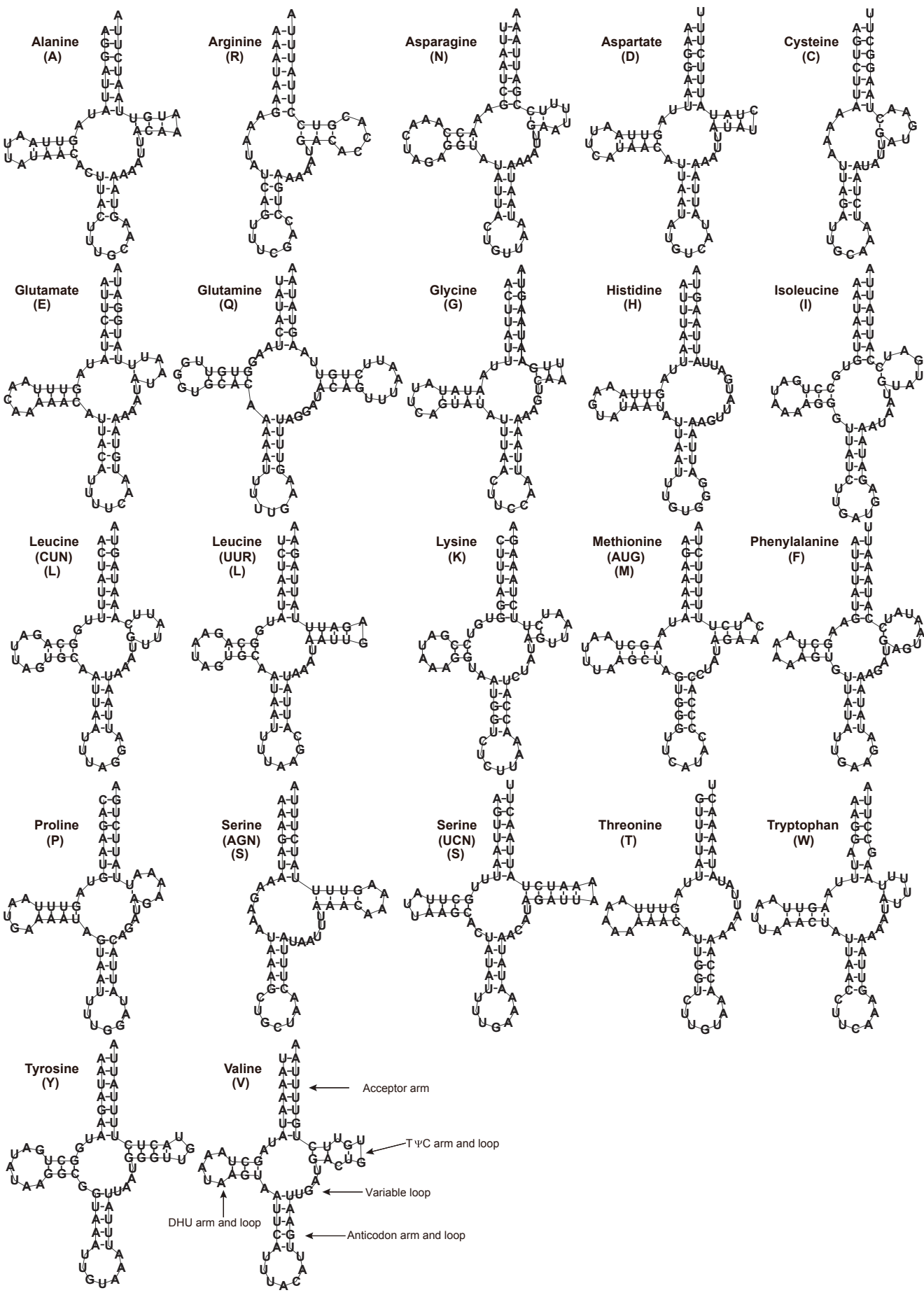

Supplement: Supplementary file 4 — Additional file 4: Secondary structures of 22 tRNAs. (PDF 1 MB) [file 12864_2014_6912_MOESM4_ESM.pdf]

**A**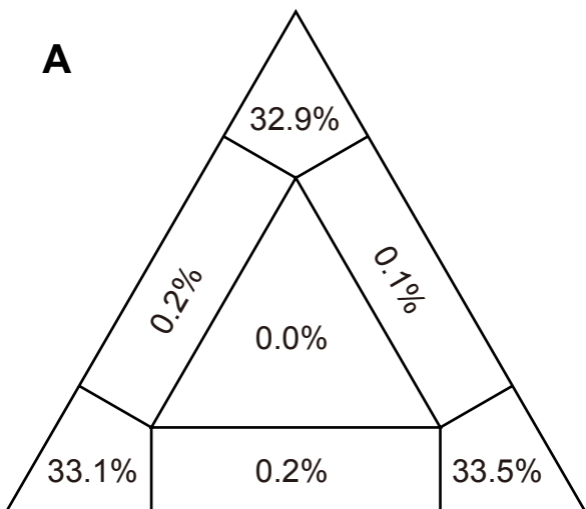**B**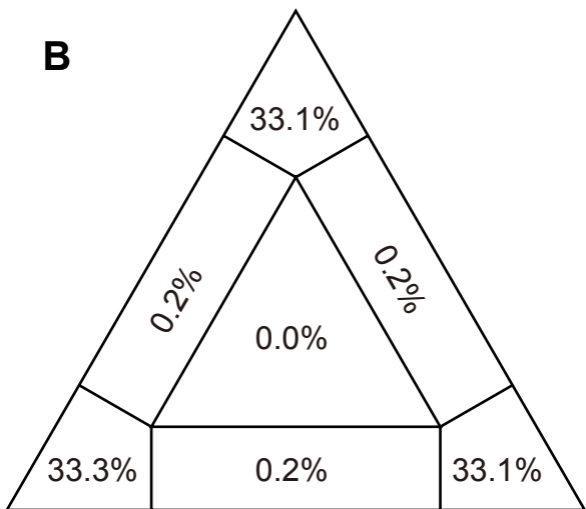**C**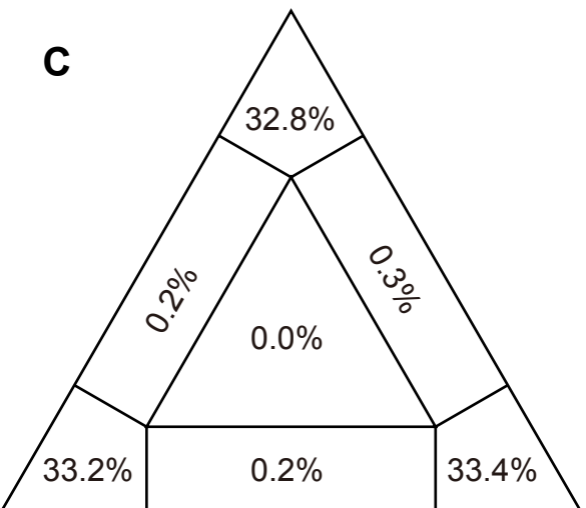

Supplement: Supplementary file 5 — Additional file 5: Likelihood map of PCG12RNA (A), PCG123RNA (B), and PCG-AA (C). The numbers indicate the percentage of dots. (PDF 262 KB) [file 12864_2014_6912_MOESM5_ESM.pdf]

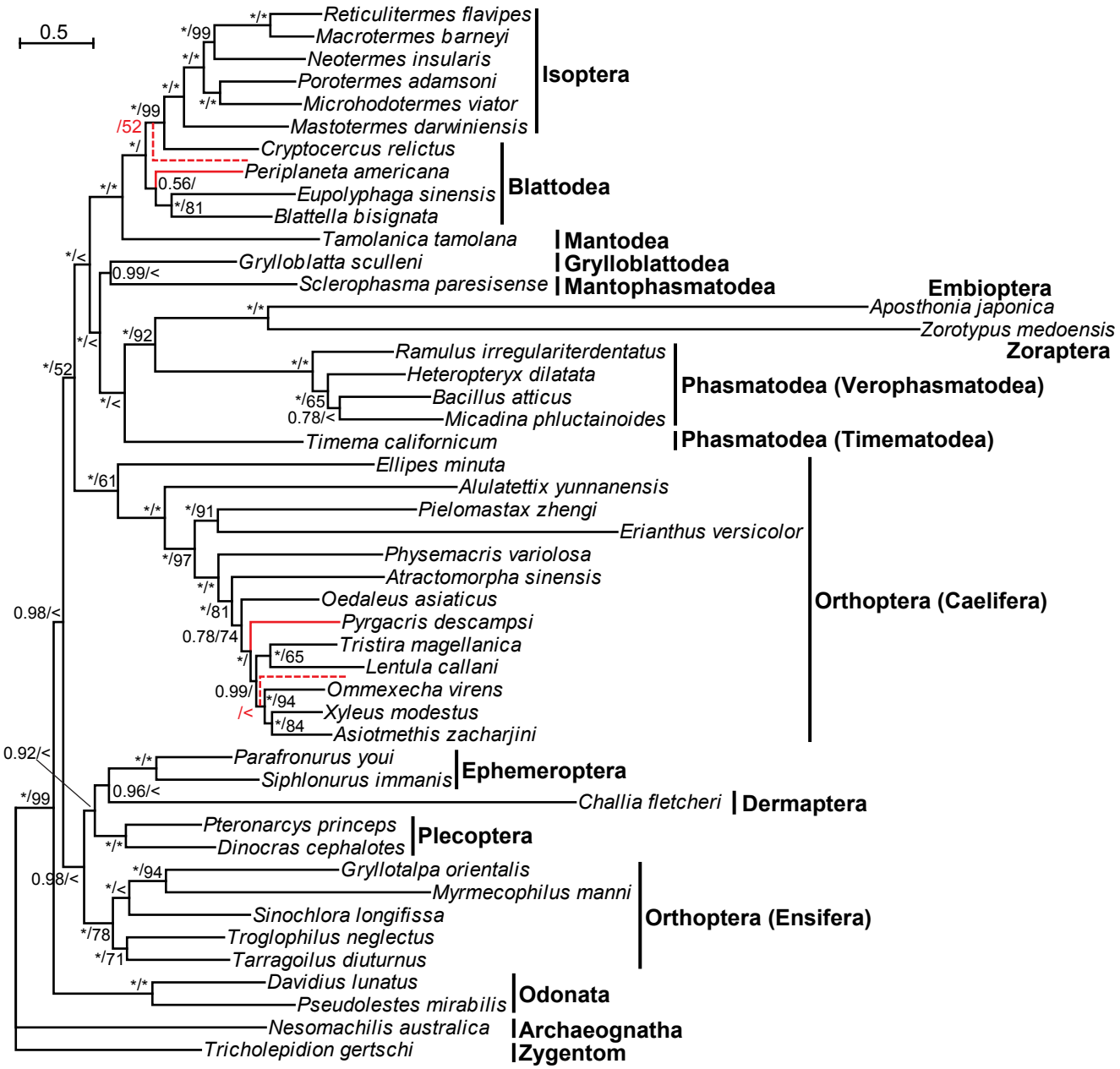

Supplement: Supplementary file 6 — Additional file 6: Bayesian phylogram of Polyneoptera based on PCG123RNA. The numbers at the nodes refer to BPP and BS values. Nodal support values of 1.00 for BPP or 100% for BS are represented by “*”; BS values below 50% are represented by “ < ”. The solid red branches are not supported by the ML method; their positions under the ML method are indicated by dashed red branches with BS values shown around. The dashed red branches are not scaled to their lengths. (PDF 444 KB) [file 12864_2014_6912_MOESM6_ESM.pdf]

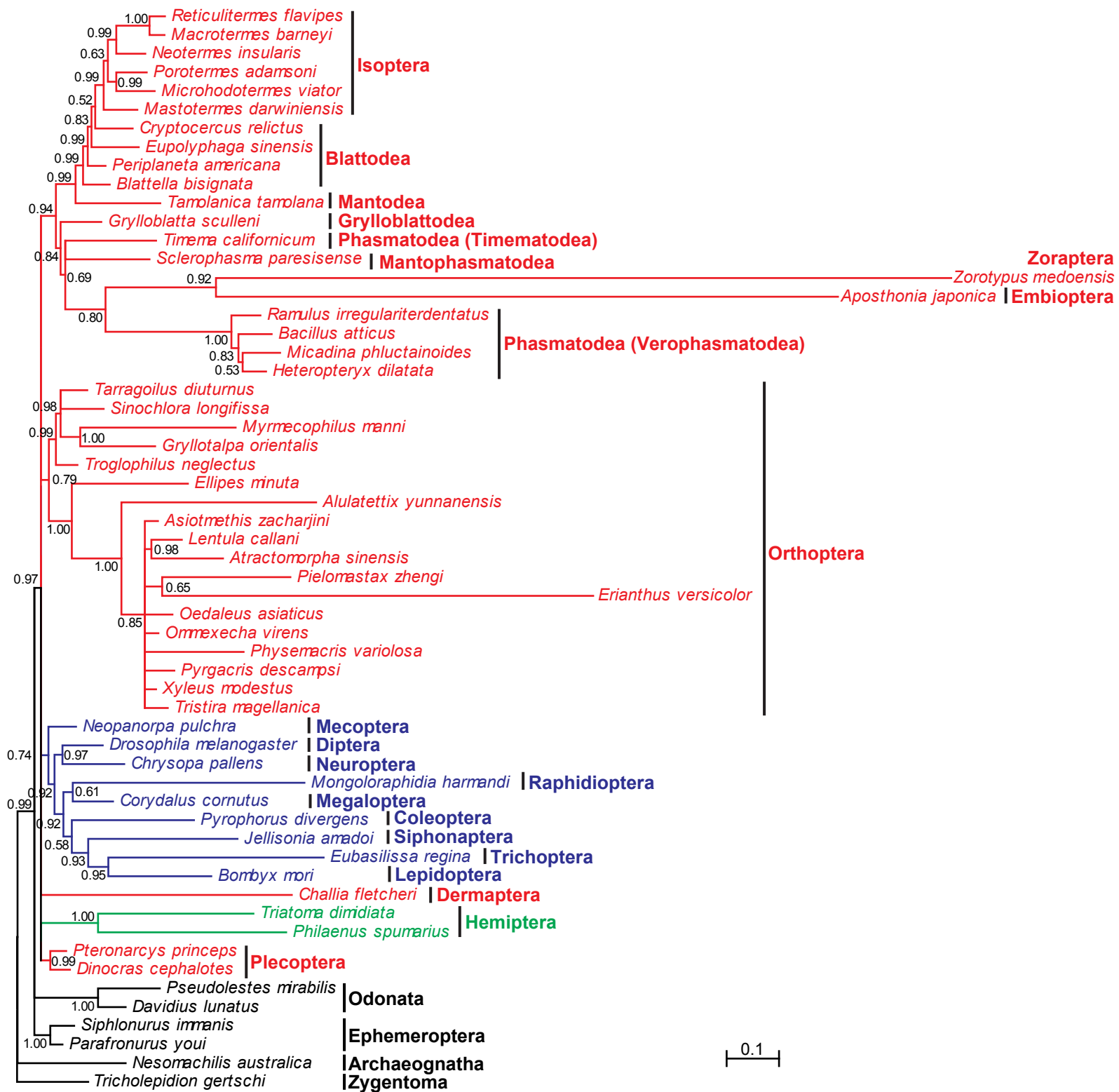

Supplement: Supplementary file 8 — Additional file 8: Bayesian phylogram inferred from concatenated protein sequences of Insecta (Insecta-AA). Branches with BPP values below 50% are collapsed. For visual clarity, Polyneoptera, Acercaria, and Holometabola are marked with red, green, and blue colors, respectively. (PDF 453 KB) [file 12864_2014_6912_MOESM8_ESM.pdf]
